# *Bartonella quintana* in Body Lice and Head Lice from Homeless Persons, San Francisco, California, USA

**DOI:** 10.3201/eid1506.090054

**Published:** 2009-06

**Authors:** Denise L. Bonilla, Hidenori Kabeya, Jennifer Henn, Vicki L. Kramer, Michael Y. Kosoy

**Affiliations:** California Department of Public Health, Richmond, California, USA (D.L. Bonilla, J. Henn, V.L. Kramer); Centers for Disease Control and Prevention, Fort Collins, Colorado, USA (H. Kabeya, M.Y. Kosoy); Nihon University, Tokyo, Japan (H. Kabeya); Napa County Health and Human Services, Napa, California (J. Henn)

**Keywords:** Bartonella, homeless persons, body lice, head lice, trench fever, vector-borne infections, California, USA, bacteria, research

## Abstract

*Bartonella quintana* is a bacterium that causes trench fever in humans. Past reports have shown *Bartonella* spp. infections in homeless populations in San Francisco, California, USA. The California Department of Public Health in collaboration with San Francisco Project Homeless Connect initiated a program in 2007 to collect lice from the homeless to test for *B. quintana* and to educate the homeless and their caregivers on prevention and control of louse-borne disease. During 2007–2008, 33.3% of body lice–infested persons and 25% of head lice–infested persons had lice pools infected with *B*. *quintana* strain Fuller. Further work is needed to examine how homeless persons acquire lice and determine the risk for illness to persons infested with *B*. *quintana*–infected lice.

## Abstract 2

Persons with lice may be at increased risk for infection with this bacterium.

The human body louse and human head louse are generally recognized as 2 subspecies of *Pediculus humanus (P*. *h*. *humanus* and *P*. *h*. *capitis*, respectively) that have distinct ecologic preferences (*1*). However, a recent genetic analysis was not able to show any differences between these 2 subspecies (*2*). The human body louse is a small, parasitic insect that lives on the body and in the clothing or bedding of its human host. Body lice feed only on human blood. In the United States, body lice infestations usually are found only on persons who do not have access to clean changes of clothes or bathing facilities (e.g., the homeless population).

Head lice also feed only on human blood and are found on the head. Head lice infestations occur most often in school children and may also occur in the homeless population where they may be transferred by pillow cases, hats, and combs. Body and head lice are morphologically indistinguishable by the unaided human eye. Body lice are most reliably differentiated from head lice by their presence on clothing or on parts of the body other than the head or neck. These lice spend most of their time on the clothing of an infested person, visiting the body up to 5 times a day to feed. The eggs (called nits) of body lice are cemented to clothing fibers and seams or, occasionally, to body hairs (*3*,*4*).

In addition to causing discomfort and irritation, body lice can transmit disease-causing pathogens. *Bartonella quintana* is a bacterium transmitted through body lice feces that are scratched into the skin by the host. This organism can cause trench fever, endocarditis, bacillary angiomatosis, peliosis, and chronic bacteremia in infected humans (*3*). Since 1992, *B*. *quintana* has been recognized as a reemerging infection in homeless populations in the United States and Europe, as well as an opportunistic pathogen in patients with AIDS (*5*). Infection with *B*. *quintana* can cause prolonged disability in immunocompetent persons and can be life-threatening in immunodeficient patients.

Studies of homeless persons seeking medical care in clinics and hospitals in the United States and France have found that 2%–20% of persons tested had antibodies against *B*. *quintana* (*6*–*9*). In Tokyo, Japan, 57% of homeless patients had immunoglobulin (Ig) G titers >128 for *B*. *quintana* (*10*). A study in Marseille, France, found that 14% (10/71) of homeless patients who came to a hospital emergency department had blood cultures positive for *B*. *quintana* (*11*). In 1990, physicians in the San Francisco, California, Bay area recognized the link between *Bartonella* spp. infections and bacillary angiomatosis (*12*,*13*) and bacillary peliosis hepatis (*14*). A subsequent study by Koehler et al. documented the occurrence of bacillary angiomatosis in 49 patients seen over 8 years (*15*). All patients in this study were infected with either *B*. *quintana* or *B*. *henselae* (the agent of cat-scratch disease), most case-patients were immunocompromised (92% had HIV infection), and *B*. *quintana* infection was associated with homelessness and body lice infestation. In a subsequent study of HIV-positive patients with fever in San Francisco, Koehler and others found that 18% of 382 patients were positive for *Bartonella* spp. (*16*).

The human body louse is currently thought to play a role in the transmission of *B*. *quintana* among homeless persons, much as it did during the epidemics of trench fever that occurred during World Wars I and II (*3*). In the aforementioned study in Marseille, France, in 1999, body lice from 3 (20%) of 15 homeless patients were positive for *B*. *quintana* by PCR (*11*). In Tokyo, Sasaki et al. examined clothing from 12 homeless persons for body lice (*17*). These authors found that lice from 2 (16.7%) of 12 homeless persons were positive for *B*. *quintana* by PCR (*17*). Furthermore, evidence now indicates that head lice may be involved in the transmission cycle of *B*. *quintana* (*18*). Homeless populations in urban areas in northern California are vulnerable to body lice infestation and may be at risk for *B*. *quintana* infection. We studied whether body and head lice from homeless populations in a northern California city are carrying *B*. *quintana* or other pathogenic *Bartonella* spp.

## Materials and Methods

In 2007 and 2008, staff from the Vector-borne Diseases Section of the California Department of Public Health (CDPH) participated in San Francisco’s Project Homeless Connect (SFPHC). Under the auspices of SFPHC medical services, hair, body, and clothing of homeless persons were inspected for lice. Any lice found on the head with the presence of nits were considered to be head lice. Any lice on the body or clothing were considered to be body lice. Most infested persons self-referred directly to the CDPH booth at this event, with the exception of 1 physician referral. Lice were collected by using forceps, identified, sorted by subspecies, and placed in screw-top vials filled with 95% ethanol. Only a portion of the total lice infesting a person were collected for testing. The lice were shipped to the Centers for Disease Control and Prevention (Fort Collins, CO, USA) for detection and identification of *Bartonella* spp.

Lice were pooled by host and then subspecies. Samples from hosts with >20 lice were further tested individually to obtain an estimate of *Bartonella* spp. prevalence in the lice. We tested 36 pools of body lice, 7 pools of head lice, 108 individual body lice, and 4 individual head lice. Individual or pooled (2–20 lice/pool) samples were suspended in 250 μL of sterile phosphate-buffered saline and homogenized in an MM300 mixer (Retsch, Newtown, PA, USA) for 8 min. DNA was extracted from the homogenates by using a Mini Kit (QIAGEN, Valencia, CA, USA) and the Blood and Body Fluid Spin Protocol according to the manufacturer’s protocol with a few minor changes. A PCR was performed in 20 μL of the mixtures containing 4–20 ng of the extracted DNA, 20 μL of 2× Ampdirect Plus, 0.5 U of Ex *Taq* Hot Start Version (Takara Bio, Otsu, Japan), and 1 pmol of each primer. *Bartonella* DNA was amplified by using *gltA* (citrate synthase gene) and *ftsZ* (cell division protein gene) primers as reported (*19*,*20*) in a thermalcycler (iCycler; BioRad, Hercules, CA, USA). A strain of *B*. *washoensis* was used as a positive control, and sterile deionized water was used as a negative control. Using gel electrophoresis on a 2% agarose gel, we examined the PCR products for 900-bp (*ftsZ*) and 380–400-bp (*gltA*) fragments.

The PCR amplicon of each gene was purified by using a QIA quick PCR purification kit (QIAGEN). Direct DNA sequencing of the purified PCR amplicons was conducted by using the BigDye Terminator Cycle Sequencing Ready Reaction Kit (Applied Biosystems, Foster City, CA, USA) with the specific primers described above on a Model 3130X Genetic Analyzer (Applied Biosystems). Sequence data of each gene were aligned and compared with type strains of *Bartonella* spp. in GenBank by using MegAlign software (DNA Star Inc., Madison, WI, USA).

## Results

In 2007 and 2008, 138 homeless persons had consultations at the CDPH booth at the SFPHC event. Of these persons, CDPH staff observed 33 persons with body lice infestations (23.9%) and 624 lice were collected (mean 18.9 lice/infested host). Head lice infestations were detected in 12 (8.7%) persons and 70 lice were collected (mean 5.8 lice/infested host). Six persons (4.3%) had body lice and head lice infestations.

*Bartonella* DNA was detected in body lice collected from 11 (33.3%) persons (Table 1) and in head lice collected from 3 (25.0%) persons (Table 2). Nine pools of body lice (n = 2–20, mean infection rate [MIR] 5%) from 9 infested persons and 2 pools of head lice (n = 7–12, MIR 8.3%) from 1 infested person showed evidence of *Bartonella* DNA. Additional lice from persons with positive pooled samples of body lice (SFB6 and SFB24) were tested individually. Sample SFB6 had 13 (87%) of 15 lice positive for *Bartonella* DNA. Sample SFB24 had 27 (64%) of 42 lice positive for *Bartonella* DNA. One of the 4 individual head louse samples (SFH2) showed amplification of *Bartonella* DNA (Table 2).

**Table 1:** *Bartonella quintana* in body lice (1–20 lice/ sample) from homeless persons, San Francisco, California, USA

Specimen ID	Date collected	No. samples tested (no. lice)		
			No. positive samples*	
			*gltA*	*ftsZ*
SFB1	2007 Feb	1 (2)	0	0
SFB2	2007 Feb	1 (1)	0	0
SFB3	2007 Feb	1 (1)	0	0
SFB4	2007 Feb	1 (4)	0	0
SFB5	2007 Feb	1 (1)	0	0
SFB6	2007 Apr	16 (35)	14	13
SFB7	2007 Apr	1 (2)	0	0
SFB8	2007 Apr	1 (4)	0	0
SFB9	2007 Aug	3 (22)	0	0
SFB10	2007 Aug	4 (23)	0	0
SFB11	2007 Aug	1 (7)	0	0
SFB12	2007 Aug	1 (14)	0	0
SFB13	2007 Aug	1 (2)	1	1
SFB14	2007 Dec	1 (2)	0	0
SFB15	2007 Dec	1 (4)	0	0
SFB16	2007 Dec	5 (91)	1	0
SFB17	2007 Dec	2 (40)	1	1
SFB18	2007 Dec	1 (6)	1	0
SFB19	2007 Dec	1 (1)	1	0
SFB20	2007 Dec	1 (5)	0	0
SFB21	2008 Jan	1 (16)	1	0
SFB22	2008 Jan	2 (21)	1	0
SFB23	2008 Jan	1 (2)	0	0
SFB24	2008 Jan	43 (62)	26	24
SFB25	2008 Jan	6 (25)	0	0
SFB26	2008 Jan	1 (3)	1	0
SFB27	2008 Jan	8 (27)	1	0
SFB72	2008 Jun	6 (25)	0	0
SFB73	2008 Jun	7 (7)	0	0
SFB74	2008 Jun	2 (2)	0	0
SFB76	2008 Jun	9 (85)	0	0
SFB77	2008 Jun	10 (10)	0	0
SFB78	2008 Jun	12 (12)	0	0

*gltA, citrate synthase gene; *ftsZ*, cell division protein gene.

**Table 2:** *Bartonella quintana* in head lice (1–20 lice/sample) from homeless persons, San Francisco, California, USA

Specimen ID	Date collected	No samples tested (no. lice)	No. positive samples*	
			*gltA*	*ftsZ*
SFH1	2007 Feb	1 (7)	1	0
SFH2	2007 Apr	1 (1)	1	1
SFH3	2007 Aug	2 (32)	1	0
SFH4	2007 Dec	1 (15)	0	0
SFH5	2007 Dec	1 (2)	0	0
SFH6	2007 Dec	1 (2)	0	0
SFH7	2007 Dec	1 (4)	0	0
SFH8	2008 Jan	1 (1)	0	0
SFH75	2008 Jun	2 (2)	0	0
SFH79	2008 Jun	1 (1)	0	0
SFH80	2008 Jun	2 (2)	0	0
SFH81	2008 Jun	1 (1)	0	0

*gltA, citrate synthase gene; *ftsZ*, cell division protein gene.

Host sample SFB16 showed no amplification of *Bartonella* DNA in its original test but when an additional 3 pools of 20 lice and 11 individual lice were tested, 1 pool of 20 lice was positive. This host had a massive body louse infestation; 91 lice were collected from his clothing. Host sample SFB27 was also negative in its first test of a pool of 20 lice; 7 additional lice tested afterwards produced a single detection of *B*. *quintana* DNA in a body louse (14%).

Samples from 1 person who was co-infested with body lice and head lice were positive for *Bartonella* DNA by the *gltA* gene PCR (SFB17, 1 pool of 20 lice) in body lice, but not in the head lice pool (SFH7, n = 4). Samples from another co-infested person were negative for *Bartonella* DNA in 1 pool of 5 body lice (SFB10). *Bartonella* DNA was detected in a pool of 12 head lice (SFH3, MIR 8.3%) (Tables 1, 2).

Thirteen (86.7%) and 12 (80.0%) body lice from host SFB6 had *Bartonella* DNA amplification by *gltA* and *ftsZ*, respectively. Twenty-five (59.5%) and 23 (54.8%) of individual body lice from host SFB24 had *Bartonella* DNA amplification by *gltA* and *ftsZ*, respectively. Two samples from hosts SFB6 and SFB24 were sequenced and found to be identical with *B*. *quintana* type strain Fuller for both genes. Host sample SFB13 had *Bartonella* DNA amplification for both genes, and showed a sequence identical to *B*. *quintana* type strain Fuller for the *gltA* product and 99.9% homology to the same type strain for the *ftsZ* product. One of the individual head lice samples, SFH2, showed positive amplification of the *ftsZ* and *gltA* genes. Sequencing showed that this sample was *B*. *quintana* type strain Fuller for the *gltA* product.

## Discussion

Our study has shown that homeless persons in the San Francisco Bay area have body and head lice that harbor *B*. *quintana* type strain Fuller. Prevalence of *B*. *quintana* in body lice from homeless persons (33.3%) in our study was slightly higher than the prevalence reported by Sasaki et al. in Tokyo, where body lice in 2 (16.7%) of 12 homeless persons were infected with *B*. *quintana* (*17*). Furthermore, similar prevalence of *B*. *quintana* infection in body lice has been reported from Russia (12.3%) (*21*) and Marseille, France (20%) (*11*).

Although Sasaki et al. detected *B*. *quintana* DNA in head lice by using molecular detection methods, their samples came from children in Nepal who also had body lice (*18*). However, there is no strong evidence that head lice are vectors of this organism between human hosts. Moreover, Fournier and others tested 143 head lice from schoolchildren from 8 countries and found no *B*. *quintana* (*22*) We have detected *B*. *quintana* in head lice from persons without a known concurrent body louse infestation. Further work is needed to examine how homeless persons acquire lice and which groups may be predisposed to louse infestation and *B*. *quintana* infection.
